# Evaluation of visual and auditory reaction time, pain, and hand grip strength performance before and after conventional physiotherapy in patients with herniated cervical intervertebral disc with radiculopathy

**DOI:** 10.14744/nci.2020.15821

**Published:** 2021-12-31

**Authors:** Deniz Senol, Fatma Kizilay, Seyma Toy, Rukiye Ciftci, Yuksel Ersoy

**Affiliations:** 1. Department of Anatomy, Duzce University Faculty of Medicine, Duzce, Turkey; 2. Department of Physical Therapy and Rehabilitation, Inonu University Faculty of Health Sciences, Malatya, Turkey; 3. Department of Anatomy, Karabuk University Faculty of Medicine, Karabuk, Turkey; 4. Department of Physical Medicine and Rehabilitation, Inonu University Turgut Ozal Medical Center, Malatya, Turkey; 5. Department of Physical Medicine and Rehabilitation, Inonu University Faculty of Medicine, Malatya, Turkey

**Keywords:** Cervical disk herniation, cervical radiculopathy, reaction time

## Abstract

**Objective:**

Herniated cervical intervertebral disc (cervical disc herniation [CDH]) with radiculopathy is known to occur in seven or eight out of 100 people worldwide. This disease causes movement limitation, loss of strength, and pain of upper extremity. The aim of this study is the effect of conventional physiotherapy agents on predetermined parameters in patients with cervical radiculopathy and to compare the results with healthy controls.

**Methods:**

A total of 102 patients with CDH with radiculopathy and 98 healthy controls were included in the study. Visual reaction time (VRT) and auditory reaction time (ART) measurements were evaluated with reaction timer, while the pain was assessed with visual analog scale (VAS) and handgrip strength (HGS) assessed with hand dynamometer, respectively. Conventional physiotherapy (transcutaneous electrical nerve stimulation, hot pack application, and therapeutic ultrasound) agents were applied 5 days/week for 3 weeks as treatment protocol.

**Results:**

As a result, VAS, VRT, and ART scores were significantly decreased, and HGS scores increased significantly in both female and male patients post-treatment measures (p<0.05). There was no statistically significant difference between patient group and control group measurements in post-treatment evaluations (p>0.05).

**Conclusion:**

This study presents the conclusion to literature that conventional physiotherapy agents have the effect of decreasing pain and regaining motor function and also a therapeutic effect on VRT and ART in the treatment of patients with CDH with radiculopathy.

**C**hronic pain is an emotional, effective, and undesired situation which negatively influences daily life activities and quality of life. In chronic pain grading, neck aches come the second after backaches. One out of three people in the general population complains about neck pain that develops due to various reasons in some part of their lives. One of the most common reasons for neck pain is cervical disc herniation (CDH) [1].

CDH frequently causes radiculopathy which includes motor and reflex changes in addition to paresthesia and pain spreading to arm accompanied with neck ache [2]. The prevalence of CDH causing radiculopathy is 0.35%, while its incidence is annually 83.2/100.000 individuals [3]. Men have been reported to be more affected than women [4]. In the presence of cervical radiculopathy, the classical clinical picture is characterized with pain spreading from the neck to the arm and fingers including the involved dermatomes; paresthesia repressing musculotendinous reflexes in the arm and hand areas; emotional discomfort; and motor weakness occurring with dermatome/myotome injury [4].

In the presence of radiculopathy, decreased reflexes and pain limit the daily activities or sportive performance of the patient. At this point, strength assessment, reaction time, and level of pain measurement will be of value in measuring the efficiency of the treatment applied. While strength is the primary motor characteristic required for movement performance, the reaction time is an inherent characteristic which determines the first muscular reaction of a person to a stimulant or the time that passes to move [5]. In the treatment of patients with CDH, treatment options are conservative therapy, rest, neck collar, anti-inflammatory drugs, transformational steroid injection, and surgical treatment [2]. Conventional physiotherapy agents are used as conservative treatment options which are used frequently in chronic pain treatment, and their efficiency is well-documented [1, 6, 7]. Transcutaneous electrical nerve stimulation (TENS), hot pack application and therapeutic ultrasound, low-level laser therapy, and pulsed electromagnetic fields are widely used in the conventional treatment [8]. Improvement in the pain and functionality levels of patients as a result of these therapies has frequently been reported [1, 4, 9]. However, evidence for its effect on reaction time seems to be very insufficient [10]. However, with the decrease in findings such as pain and paresthesia as a result of therapy, motor performance characteristics such as strength and reaction time will be shown more objectively.

**Highlight key points**

• CDH with radiculopathy patients, pain decreased and HGS increased as a result of conventional physiotherapy.
• A decrease in VRT and ART scores, which had never been evaluated before was found.
• Despite innovative treatment alternatives, conventional physiotherapy agents are still frequently used and effective methods that should be preferred.

The hypothesis of this study is to determine whether visual reaction time (VRT) and auditory reaction time (ART) can be improved by taking advantage of the effect of conventional physiotherapy on pain. From this point of view, the purpose of the present study is to show the effects of conventional physiotherapy agents on pain, VRT, ART, and handgrip strength (HGS) in patients with CDH with radiculopathy.

## Materials and Methods

The study was conducted with the approved permission of Malatya Clinical Researches Ethical Board (2018/23). Study was conducted from September 2018 to September 2019 in a Malatya hospital, in the department of physical medicine and rehabilitation.

### Selection and Description of Participants

The sample size was automatically calculated on the G-power package program. In the sample size calculation, type I error 0.05 and type II error 0.20 were taken. The strength of the test was determined to be 0.8. It was determined that the minimum number of subjects should be 41 male and 41 female in patient group and 41 male and 41 female in control group to make a difference of 2.96 units in the mean difference value between the two groups for hand grip strength [11]. In the cervical magnetic resonance imaging examination performed in the past 6 months, patients with at least one spinal level of cervical radiculopathy were included in the study.

One hundred and two patients of CDH with radiculopathy between the ages of 30 and 65 and 98 healthy controls between the same age ranges participated to the study. Patients who had not taken any analgesic drug within the past 72 h and who had been diagnosed as radiculopathy (with complain of neck pain irradiation to the arm and fingers corresponding to the dermatomes, paresthesias in arm and hand in conjunction with diminished muscle tendon reflexes, sensory disturbances, and/or motor weakness with dermatomal/myotomal distribution) [4] were included the study.

**Figure 1.**
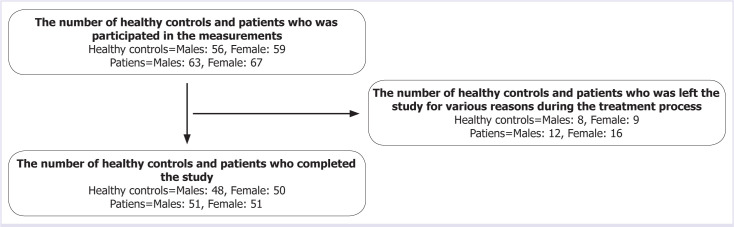
The flowchart of the study.

The patients who had musculoskeletal system disease that could influence motor function, those who had headache in addition to neck ache, those who had pains such as extremity pain, and those who had received conventional therapy to neck area during the past year or who had a surgery history related with the neck area were excluded from the study. All the patients who participated in the study were informed about the study and they were asked to read and sign an informed consent form. The study protocol was conducted according to the Declaration of Helsinki. The flowchart of the study is shown in Figure 1.

### Technical Information

All measurements and treatment protocol were performed and recorded by the same physiotherapist with the same physician in physical medicine and rehabilitation department.

### Reaction Time Measurement

VRT and ART measurements of the patients in the study were conducted with Hubbard Scientific Reaction Timer (Model: 6027, USA). Reaction timer device can give two different warnings as visual (light) and auditory (sound). The patient was asked to respond by pressing the pedal of the reaction timer device to the visual and auditory warning that given by clinician. Ten trials were taken from each patient for sound and light stimuli. The first five were accepted as exercise, and the average of the last five trials was determined as reaction time [12]. The first measurements were taken before the patients started the therapy, while the second measurements were taken immediately after the therapy ended. Measurement results specified as millisecond (ms).

### HGS Measurement

For HGS measurement, Jamar hand dynamometer (Lafayette Instrument Company, USA), which is recommended by American Society of Hand Therapists (ASHT) and which has been found to have high validity and reliability in many studies and thus has been accepted as the gold standard, was used. HGS measurement was performed in sitting position, the shoulder in adduction and neutral rotation, the elbow with 90° flexion, the forearm in mid-rotation and supported, and the wrist neutral, which is the recommended standard position by ASHT [13]. Measurement results specified as kilogram (kg).

### Pain Analysis

Pain analysis was assessed with visual analog scale (VAS). The patients were asked to mark their level of pain on a 10 cm ruler on the right and left ends of which were written 0=no pain and 10=unbearable pain, respectively. The marked distance was measured from the left end of the ruler [14].

### Treatment Protocol

TENS was applied to the cervical region in all patients 20 min a day, 5 days a week for 3 weeks with Compex Theta MI Pro. Furthermore, 20 min of hot pack application was used as a superficial heat agent. Therapeutic ultrasound, which was used as a deep heat agent in the patients, was applied with a frequency of 1 MHz and 1.5 watts/cm^2^ intensity for 10 min [15]. Post-treatment evaluations had been applied immediately after 3 weeks of treatment.

### Statistical Analysis

Normality distribution of the data was examined with Kolmogorov–Smirnov test and it was found that the data were not normally distributed. Mann–Whitney U analysis was conducted for comparisons of age, weight, and height between patients and control group. The changes in pre-treatment, post-treatment, and control groups VRT, ART, R-HGS, and L-HGS were measured with Kruskal–Wallis H-test. Mann–Whitney U-test was applied as post hoc test. Adjusted significant values were given in the analysis results. VAS scores were measured with Wilcoxon matched pairs test. The data which were not normally distributed were shown with median and minimum (min) and maximum (max) values. P<0.05 was considered as statistically significant. For statistical analysis, SPSS Statistics 22.0 (IBM Corp., Armonk, NY, USA) for Windows package program was used.

## Results

Fifty-one male patients with a median age of 54 years (min 30 age–max 65 age) and 51 female patients with a median age of 55 years (min 21 age–max 64 age) were included in the study. The control group included 48 males with a median age of 51 years (min 33–max 65) and 50 female healthy volunteers with a median age of 52 years (min 26–max 65). As a result of the Mann–Whitney U analysis conducted, no statistically significant difference was found between the ages, height, weights, and BMI parameters of male and female patients in both patient and control groups in the study (p>0.05) (Table 1).

**Table 1.** Median (min-max) values and Mann–Whitney U analysis results of age, weight, and height variables of male and female patients and control groups

Sex	Variables	Patients	Healty control	p
Male	Age (year)	54 (30–65)	51 (33–67)	0.211
	Weight (kg)	80 (58–110)	79 (60–113)	0.326
	Height (cm)	175 (165–185)	174 (161–190)	0.240
	BMI (kg/m**2**)	26.1 (21.4–32.3)	25.9 (24–31.3)	0.411
Female	Age (year)	55 (21–64)	52 (26–66)	0.098
	Weight (kg)	73 (45–105)	70 (47–103)	0.086
	Height (cm)	157 (145–178)	160 (147–180)	0.091
	BMI (kg/m**2**)	28.6 (21.4–33.2)	27.3 (22.3–31.6)	0.087

BMI: Body mass index.

For male participants, there was a statistically significant difference between pre-treatment, post-treatment, and control measurements in terms of VRT, ART, R-HGS, and L-HGS parameters (p<0.05) (Table 2).

**Table 2.** Pre and after the treatment VRT, ART, R-HGS, and L-HGS median (min-max) values of male patients and control groups

Parameter	Patients		Healty		p
	Pre-treatment	Post-treatment	Control	
VRT (ms)	31.6 (21–73.3)		22.3 (11.6–46.3)	21 (13–41)	<0.001
ART (ms)	34.6 (19.6–85)		24.3 (13–40)	22.3 (12–40)	<0.001
R-HGS (kg)	65 (30–90)		74 (30–95)	76 (42–96)	0.001
L-HGS (kg)	60 (30–84)		72 (32–92)	76 (44–94)	<0.001

VRT: Visual reaction time; ART: Auditory reaction time; R-HGS: Right-hand grip strength; L-HGS: Left-Hand Grip Strength.

For female participants, there was a statistically significant difference between pre-treatment, post-treatment, and control measurements in terms of VRT, ART, R-HGS, and L-HGS parameters (p<0.05) (Table 3).

**Table 3.** Pre and after the treatment VRT, ART, R-HGS, and L-HGS median (min-max) values of female patients and control groups

Parameter	Patients		Healty		p
	Pre-treatment	Post-treatment	Controls	
VRT (ms)	37 (18.6–83.6)	24.6 (15–40.6)	22.4 (16–42.6)	<0.001
ART (ms)	31.3 (19–71.6)	26.6 (12–48.67)	23 (12–50)	<0.001
R-HGS (kg)	35 (10–70)	45 (15–77)	48 (22–80)	<0.001
L-HGS (kg)	35 (10–65)	42 (17–70)	48 (16–78)	<0.001

VRT: Visual reaction time; ART: Auditory reaction time, R-HGS: Right-hand grip strength, L-HGS: Left-hand grip strength.

The Mann–Whitney U post hoc test shows the difference between the pre-treatment, post-treatment, and control measurements. Accordingly, the difference of pre-post-treatment measurements and pre-treatment-control measurements was statistically significant (p<0.05). The difference in post-treatment-control group measurements was not statistically significant (p>0.05), (Table 4). In this case, it has been revealed that conventional physiotherapy applied to patients improves VRT, ART, and HGS values.

**Table 4.** Mann–Whitney U-test results performed as post hoc test of male and female patients

Binary comparison	Adjusted p value	
	Male	Female
Pre-treatment-post-treatment	0.032	0.036
Pre-treatment-control	0.028	0.027
Post-treatment-control	0.231	0.141

For the VAS variable, the difference in pre-post-treatment values was found statistically significant in both women and men in the patient group (p<0.05) (Table 5).

**Table 5.** Pre-treatment and post-treatment median (min-max) values of VAS and test results of male and female patients

Sex	Pre-treatment	Post-treatment	
	Median (min-max)	Median (min-max)	p
Male	7 (5–9)	4 (1–7)	<0.001
Female	7 (4–9)	4 (1–7)	<0.001

VAS: Visual analog scale.

## Discussion

A total of 102, 51 female and 51 male, CDH with radiculopathy patients were assessed in this study and as a result of the 15 sessions conventional therapy, decrease in pain, increase in HGS, and a statistically significant decrease in VRT and ART values was found.

Neck pain is an essential musculoskeletal system disease the prevalence of which can change from 16.7% to 5.1% in the adult population and which causes social and economic losses by decreasing individuals’ quality of life [16, 17]. CDH is reported as one of the leading factors causing neck ache [1].

A literature review which was conducted showed that post-treatment effects of conventional physiotherapy in patients with CDH were well-reported [1, 4, 6, 9, 17]. It was found that evidence about the effect of conventional physiotherapy agents, which are found to have positive effects on pain, motor performance, functional attainments, and daily life activities in literature frequently used in clinics, on VRT and ART in patients with CDH with radiculopathy, were very insufficient.

When situations such as pain paresthesia resulting from cervical radiculopathy disappear, the individual can show his/her real performance. In this study, the pain that appeared to be reduced on post-treatment evaluations probably led to the emergence of the muscle strength of the individuals. This situation was reflected in post-treatment results as a significant increase in HGS scores. It is thought that radiculopathy with disc hernia can also influence reaction time due to its adverse effects on individuals’ muscle strength and motor performance [10]. HGS is an essential prerequisite for the proper performance of the upper limb and checking the normal activities of hand function also plays a significant role in upper extremity assessment. Faisal et al. [18] reported that in patients with unilateral cervical radiculopathy, the unaffected side had more HGS and hand function when compared with the affected side.

In their study, Egwu et al. [19] stated that they found HGS much lower in patients with cervical spondylosis when compared with healthy individuals in the control group. Furthermore, no statistically significant difference was found between the dominant and non-dominant hand in terms of HGS. When pre-treatment, post-treatment, and control values were compared, a statistically significant improvement was found in HGS values of patients in this study.

Reaction time is also influenced by pain. In terms of analyzing the effectiveness of conventional physiotherapy agents on the musculoskeletal system, the improvement in reaction time can be an important indicator [20]. In this study, statistically significant improvement was found in post-treatment VRT and ART scores when compared with the pre-treatment scores. No significant difference between post-treatment and control measurements shows that VRT, ART, and HGS values have reached the level of measurements in the healthy group. As a result of this study, the motor component of reaction time, which is known to be the first response to visual and auditory stimulus, has positively affected the motor component, and it has created a statistically significant positive effect on VRT and ART scores. Studies which report that conventional physiotherapy agents have a direct influence on pain mechanisms and motor function support the results of the present study [21]. Degenerative changes associated with cervical spondylosis results in disturbed motor control, eventually leading to decreased cervicocephalic kinesthetic sensibility and reduced reaction or response to sudden, spontaneous stimuli [22]. Lechner et al. [23] conducted driving reaction time study in patients with a CDH and reported that VAS decreased following surgical intervention. Sargent et al. [24] found that patients with cervical radiculopathy had longer reaction time when compared with healthy individuals. In their study, Shankar et al. [25] found that patients with cervical spondylosis had longer reaction time when compared with healthy individuals.

While the association between chronic backache and the lower extremity reaction time is one of the studied subjects in the literature, the number of studies on the association between CDH patients and VRT, ART, pain, and muscle strength is very limited [10, 26]. In the present study, while statistically significant increase was found in post-treatment HGS values of the patients, a statistically significant decrease was found in pain. It is believed that increased HGS values with significant pain reduction have a direct effect on reaction time.

## Conclusions

These results support that despite innovative treatment alternatives, conventional physiotherapy agents continue to be used frequently and they are effective methods that should be preferred in the treatment of patients of CDH with radiculopathy in terms of causing a decrease in the pain which has a negative influence on the patients’ daily life activities, regaining motoric function and improving reaction time.

Difficulty of patients coming to attend 3-week physiotherapy sessions due to their living conditions limited the work and created limitation. Although the fact that this study is the first in this area creates a limitation for the discussion section, it will form the basis of new studies in this area.
